# The Use of Nutritional Interventions to Enhance Genomic Stability in Mice and Delay Aging

**DOI:** 10.3390/nu18020246

**Published:** 2026-01-13

**Authors:** Ivar van Galen, Jan H. J. Hoeijmakers, Wilbert P. Vermeij

**Affiliations:** 1 Princess Máxima Center for Pediatric Oncology, 3584 CS Utrecht, The Netherlands; ivar.vangalen@maastrichtuniversity.nl (I.v.G.); j.hoeijmakers@erasmusmc.nl (J.H.J.H.); 2 Oncode Institute, 3521 AL Utrecht, The Netherlands; 3 Laboratory for Experimental Orthopedics, Department of Orthopedic Surgery, Maastricht University, 6229 ER Maastricht, The Netherlands; 4 Department of Molecular Genetics, Erasmus MC Cancer Institute, Erasmus University Medical Center Rotterdam, 3015 GD Rotterdam, The Netherlands; 5 Institute for Genome Stability in Ageing and Disease, Cologne Excellence Cluster for Cellular Stress Responses in Aging-Associated Diseases (CECAD), University of Cologne, 50931 Cologne, Germany

**Keywords:** dietary interventions, DNA damage and repair, aging

## Abstract

**Background/Objectives:** Metabolism is fundamental to all living organisms. It comprises a highly complex network of fine-tuned chemical reactions that sustain life but also generate by-products that damage cellular biomolecules, including DNA, thereby contributing to aging and disease. As metabolism can be largely modified by dietary alterations, it has the potential to positively or negatively affect health and disease. Interestingly, many aging-associated illnesses known to be influenced by diet also show a causal relation with DNA damage. As DNA keeps all instructions for life, and DNA lesions, if unrepaired, interfere with vital processes such as DNA replication and transcription, DNA damage may be an important mediator of the impact of nutrition on health and aging. **Methods:** Here, we discuss the genome-protective effects of various oral interventions in mice, aiming to elucidate which nutritional alterations lower DNA damage and promote overall health. **Results:** Our analysis covers a wide range of interventions with reported positive impacts on genomic stability, including modified diets (e.g., dietary restriction, probiotics, micronutrients, fatty acids, and hormones), NAD+ precursors (e.g., nicotinamide riboside), plant derivatives, and synthetic drugs. Among these, caloric and dietary restriction emerge as the most potent, generic modulators of DNA damage and repair processes, enhancing aspects of repair efficiency through metabolic recalibration and improved cellular resilience. Other interventions, like NAD+ precursors, activate partly similar pathways without necessitating reduced food intake. **Conclusions:** While many interventions show promise, their effects are often less pronounced or are process-specific compared to caloric or dietary restriction. Additionally, many substances lack comprehensive exploration of their genome-protective effects in mice, with often only a small number of studies examining their impact on genome stability. Moreover, the heterogeneity between studies limits direct comparison. However, the observed overlap in mechanistic effects between treatments lends credibility to their potential efficacy. Ultimately, a deeper understanding of these mechanisms could pave the way for translating these findings into, e.g., combination treatments to promote healthy aging in humans.

## 1. Introduction

Metabolism consists of an intricate network of chemical reactions that sustain life and serve as the foundation of biological functionality across all living organisms [1]. The fundamental role of metabolism extends beyond mere survival, growth, and development, as it also influences health status and disease trajectories by modulating the body’s internal environment and maintaining homeostasis in the face of exogenous stressors [2]. Dietary patterns significantly affect metabolic pathways, thereby impacting energy balance, metabolic fitness, the amount and type of reactive by-products of metabolism, and overall health [3,4,5]. Furthermore, these metabolic processes are both consequences and drivers of the aging process, and may elicit anti-aging effects, implicating both longevity and quality of life [6,7].

Many age-related chronic diseases, such as cardiovascular disorders, neurodegeneration, and cancer, share a causal process; DNA damage (DD) [8,9,10]. DD generally occurs in a stochastic manner and is highly heterogeneous, ranging from small oxidation products (e.g., 8-oxo-dG) and abasic sites (induced by spontaneous hydrolysis, occurring ~10^4^ times per cell per day), to helix-distorting bulky adducts, intrastrand and interstrand crosslinks, and protein–DNA crosslinks, and various forms of single- and double-strand breaks [11]. Each of these types of lesions is counteracted by one or more specialized DNA repair mechanisms [11]. Base excision repair (BER) corrects small, non-helix-distorting base lesions, while nucleotide excision repair (NER) and transcription-coupled repair (TCR) remove bulky adducts that distort the DNA helix and/or physically impede elongating RNA polymerases, resp. More disruptive lesions, like double-strand breaks, are repaired either by homologous recombination (HR), when the (proliferating) cell is in S-phase or G2, which restores the original sequence using the replicated intact copy as a template, or by non-homologous end joining (NHEJ), which directly ligates the broken DNA ends but is less error-free.

Not all DD is repaired perfectly, and the accumulation of DD over time, caused by both endogenous (e.g., reactive metabolic by-products) and exogenous factors, is now recognized as a major hallmark of aging (Table 1). This accumulation influences both the genetic and epigenetic landscape, disrupting cellular homeostasis and driving processes such as senescence and apoptosis, ultimately manifesting as age-related physiological decline [12,13]. Notably, progeroid DNA repair-deficient mouse models such as *Xpg*^−/−^ and *Ercc1*^Δ/−^ mice, in which DD persists, resulting in an accelerated and shortened lifespan, exhibit epigenetic alterations, transcriptional changes, systemic biomarker shifts, and multi-organ functional decline that closely mirror those observed during natural aging, supporting their use as mechanistic models of physiological aging [12,13]. However, accurately measuring physiological (i.e., low) levels of DD and reliably assessing DNA repair activity remains technically challenging (Table 1). Methods such as the comet assay require delicate handling, and many genome maintenance interventions lack robust evidence due to methodological limitations. Additionally, antibody-based γH2AX detection is widely used to quantify DNA double-strand breaks; however, these lesions are rare, and γH2AX can also mark dying cells, complicating data interpretation. Still, growing evidence suggests that environmental and lifestyle factors, particularly diet, influence the accumulation of DD.

Depending on diet, health and lifespan can be positively or negatively influenced [26]. Notably, altering metabolism through dietary interventions offers a promising approach to enhancing genomic stability. Nutritional components can either provoke or protect against DD through multiple, partially overlapping mechanisms such as (i) direct effects on DD formation or repair capacity, (ii) changes in metabolic rate and energy flux that alter the production of endogenous genotoxic stress, and (iii) enhanced cellular and organismal resilience, which could indicate, for example, improved tolerance to damage without necessarily reducing lesion burden or enhanced DD response signaling. For instance, certain nutrients act as cofactors in enzymatic reactions that repair DNA, while others may modulate gene expression and mitigate cellular stress [27]. Conversely, dietary excesses of protein, simple carbohydrates, and fat can induce metabolic stress and inflammation, thereby facilitating genomic instability [28,29,30].

The interplay between diet, metabolism, and DD is particularly critical as it represents a modifiable factor in the aging process. With the global population aging at an unprecedented rate [31], there is a pressing need to understand and harness these relationships to devise dietary strategies capable of decelerating the aging process and prolonging a healthy lifespan [32,33].

## 2. Materials and Methods

**Figure 1:**
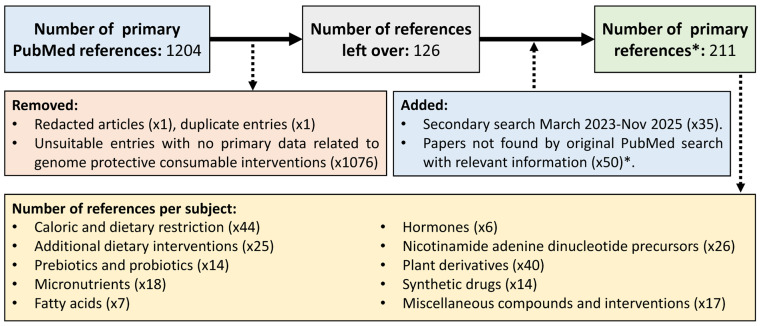
Schematic representation of the reference selection used in this review. PubMed references obtained from the query-based search were filtered based on relevance to genome-protective consumable interventions, with unsuitable and duplicate entries removed. Relevant articles identified after our primary search were subsequently added. The total number of references is highlighted per subject, while allowing for potential overlap. * excluding references exclusively used in Section 1 and Section 4.

This review focuses on various nutritional interventions aimed at reducing DD induction and/or improving repair mechanisms in the contexts of health and longevity in mice. Relevant literature published on PubMed was collected using two automated search queries using a semi-systematic approach without formal quality assessment. The first search consisted of all applicable PubMed papers (excluding pre-prints) up to 1 March 2023, which was supplemented with a second search for all papers published between 1 March 2023 and 1 November 2025 (see Supplementary Files S1–S3 for the search queries and results). Search queries included at least one primary keyword related to DNA damage and repair, one secondary keyword related to aging and health, and one tertiary keyword specific for the type of nutritional intervention, all restricted to Mus musculus (Supplementary File S1). Entries were examined in a semi-systematic review format and supplemented with articles, e.g., based on references not identified by the original search parameters. Articles were screened for relevance by assessing their inclusion of data related to DD or DD repair, and studies lacking well-defined oral treatments or involving non-edible interventions were excluded. This approach was chosen to maximize the global overview and identification of potentially relevant literature, as many studies likely contain or assessed only one type of nutritional intervention or DNA damage detection method, or looked at specific mouse models or tissue types. Although no formal quality assessment was performed, yielding a potential bias, all included studies were peer-reviewed, and articles for which authors declared a potential conflict of interest are highlighted with ‡. Ambiguities regarding these selection criteria were resolved through evaluation by a second assessor. The final dataset comprises 69 studies on dietary interventions including restrictive diets, 111 on supplements, 14 on oral medications, and 17 on other miscellaneous interventions, all with a focus on beneficial effects related to DD and its repair (Figure 1). Ultimately, this review aims to identify consumable interventions that improve genomic stability in mice and may reduce chronic diseases in humans.

## 3. Results

### 3.1. Caloric and Dietary Restriction

The oldest known and most extensively studied nutritional intervention is dietary restriction (DR), where total food intake is typically reduced by 10–40% without causing malnutrition [34,35,36]. Caloric restriction (CR) is often used synonymously, but technically refers to a reduction in caloric intake without altering total micronutrient intake, like vitamins and minerals, which is in contrast to DR, where both macro- and micronutrient composition may vary [37]. Here, we will use the term “dietary restriction” to refer to studies where total food consumption was reduced, and “caloric restriction” when this was compensated with additional micronutrients.

Both CR and DR trigger reduced energy expenditure by, e.g., lowering growth hormone, insulin-like growth factor (IGF1), thyroid hormone (receptor), mTOR, glucose, insulin levels, and body temperature [38,39]. The resulting recalibration of energy metabolism under a negative energy balance is associated with reversed age-related decline in mitochondrial activity by restoring oxidative capacity, leading to improved efficiency and redox homeostasis without increasing mitochondrial mass [38]. Consequently, oxidative damage, as measured by FpG- and Endo III-sensitive breaks in a comet assay [40], and 8-oxo-2′-deoxyguanosine (8-OHdG) oxidative biomarker levels, is lowered in various tissues, including muscle, brain, kidney, and liver [38,41]; however, in hippocampal subfields of aged wild-type C57BL/6J mice, 8-OHdG levels have been reported to increase [42]. This amelioration appeared more pronounced in post-mitotic tissues and after extended duration of the dietary intervention. Interestingly, 10 months of CR, the only period tested, demonstrated protective effects against clofibrate, an indirect liver carcinogen and oxidative stress-inducing agent specific for rodents [43], likely due to improved stress resilience and/or enhanced DNA repair.

By affecting cellular processes, 30% DR and CR are able to extend median and maximum lifespan by approximately 15–30% in wild-type mice, although considerable variation has been noted between different laboratory mouse strains [34,44]. Interestingly, these lifespan-extending effects are even more pronounced in prematurely aging DNA repair mutants [45]. In *Ercc1*^Δ/−^ and *Xpg*^−/−^ mice, which carry deficiencies in multiple DNA repair pathways, including nucleotide excision and transcription-coupled repair, and mimic features of human progeroid syndromes, DR increases median remaining lifespan by ~200% and ~80%, respectively. These increases are associated with improved tissue function and histological parameters in various organs, including muscle, liver, kidney, bone, vasculature, and most prominently, the nervous system [45,46,47,48,49,50]. As these mutant mice are genetically defective in their DNA repair capacity, their heightened response to the protective effects of DR is most logically explained by a reduced DD load. One strong piece of evidence for this comes from studies showing that DR leads to lower genome-wide DD, as reflected by reduced γH2AX foci and diminished gene length-dependent transcription stress [14,45]. Since these genetic mutants are, in part, functionally defective in DNA repair pathways and no significant back-up repair systems are known, this reduction in DD is unlikely to result from enhanced repair activity. Instead, it is more plausibly attributed to a decreased metabolic production of DD or an improved ability of cells to tolerate and manage cellular stress [46].

In addition to limiting the production of endogenous DNA-damaging agents, DR has been reported to alter DNA repair activity and the expression of related proteins. Specifically, short-term intervention (4 weeks) in 3–5-month-old wild-type C57BL/6J mice increased DNA-PK and SIRT6 levels and the efficiency of NHEJ in skin, lung, kidney, and brain [51]. Similarly, DR protected against chemical mutagenesis and improved base excision repair (BER) in the brain, liver, kidney, spleen, and testes, possibly by preventing the age-related decline in the levels and activity of key proteins, such as the BER rate-limiting enzyme DNA polymerase beta [52,53]. Conversely, BER activity in the mitochondria of kidney and brain was significantly downregulated, suggesting organelle-specific changes [53]. DR reduced the level of *Igf1*, which was inversely correlated with *Dclre1a* [54], a gene whose protein product plays an important role in degrading chemically modified DNA during ICL and DSB repair [55]. DR has also been reported to downregulate the expression of various genes related to DNA repair in skeletal muscle, including, *Ddb2*, *Rad50*, and *Polb* [39]. In contrast, DR upregulated several DNA repair pathways and related proteins in heart, liver, and brain tissue [56,57,58], suggesting tissue-specific expression differences. Furthermore, DR does not seem to influence the quantity of single-strand breaks in the brain, kidney, and liver of old wild-type animals, as measured by fluorometric analysis of DNA unwinding [59]. Together, these findings highlight the diverse effects of DR and CR on genomic stability and repair, several of which demonstrate their potential to optimize repair processes and mitigate damage in a tissue-specific manner.

The idea of improved genomic stability by DR is further supported by old preliminary studies using unscheduled DNA synthesis (UDS), which reflects a cell’s ability to perform global genome nucleotide excision repair (GG-NER) [60]. For example, UVC irradiation of freshly isolated skin cells from mice on 40% DR increased UDS by 48–65% compared to AL controls [61]. Similarly, while UVC generates transcription-blocking bulky lesions, DR reduced the age-associated decline in enzyme activity and fidelity of DNA polymerase α and β in hepatocytes [62,63]. Additionally, in wild-type C57BL/6J mice, 30% DR reduced UVB-induced skin damage, histological changes, and inflammatory responses, but refeeding 24 h after irradiation partially reversed these benefits [64]. However, it also delayed γH2AX repair, suggesting that while DR limits initial damage, it also partially appears to slow recovery, possibly by reducing proliferation and the activity of replication-dependent DNA repair mechanisms like HR and XLR [64,65]. How DR affects transcription-associated DNA repair mechanisms is yet unknown. However, we have previously reported that age-related, DD-induced, gene length-dependent transcriptional decline (GLTD) was alleviated in DNA repair-deficient mutant animals when on DR, compared to ad libitum animals. This indicates that DR reduced transcription-blocking lesions genome-wide, consistent with the idea that DR lowers the overall DD load as at least one way by which it delays systemic aging [45,49,66].

Besides UV protection, DR confers protection against multiple types of genomic insults. In wild-type C57BL/6J mice, 30% DR reduced tumor incidence at necropsy [67], likely because DR reduces genomic mutation rates [68,69]. Additionally, 30% DR has been shown to protect against toxicity induced by fine ambient particulate matter, as evidenced by reduced DD, oxidative stress markers, inflammation, and improved lung tissue morphology [70].

Concurrent with the attenuation of genomic damage and the enhancement of DNA repair pathways, DR reduces natural age-related apoptotic markers and damage-inducible transcripts in the heart, such as *Bax*, *Bad*, *Casp9*, *Casp11*, and *Gadd45a* [57]. Furthermore, long-term DR reduces senescence, inflammation, and γH2AX foci in multiple tissues and cell types, including brain, adipose, corneal epithelium, and hematopoietic stem cells (HSCs) [71,72,73,74,75], although these effects are less pronounced in short-term DR [75]. Interestingly, many of these markers remain lowered for at least three months following a return to AL feeding after nine months of 40% DR in wild-type C57BL/6J mice [76], suggesting a prolonged protective effect.

**Figure 2:**
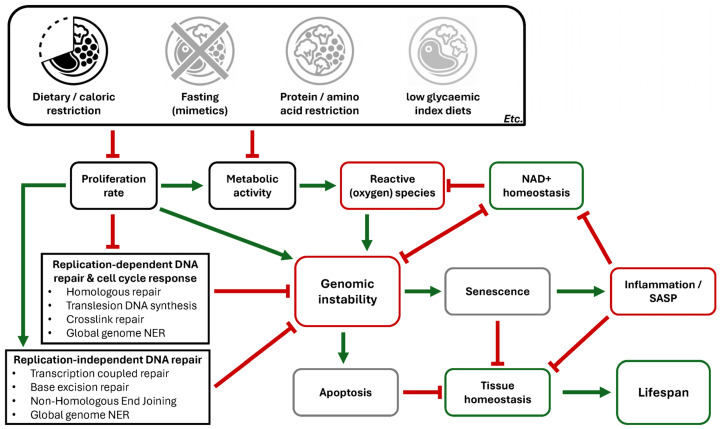
Dietary interventions influence genome integrity and aging-associated processes. Schematic overview of how dietary interventions modulate cellular metabolism and genomic maintenance. As caloric/dietary restriction yields the most robust effects, other diets are depicted in lighter tones. Interventions reduce proliferation and metabolic activity, lowering reactive oxygen species while improving replication-independent DNA repair, which together limit genomic instability. Enhanced DNA repair and reduced oxidative stress reduces apoptosis, senescence, and inflammation, contributing to improved tissue homeostasis and lifespan extension. Green arrows indicate promoting effects; red blunt lines indicate inhibition. Green and red outlines indicate positive and negative processes, respectively.

In conclusion, CR and DR exhibit a profound and multifaceted impact on genomic stability by mitigating age-related DD and enhancing the efficiency of repair pathways, thereby contributing to improved tissue function and an extended lifespan (Figure 2). The tissue- and stressor-specific protective effects further underscore the potential of these interventions as powerful modulators of aging and genomic integrity.

### 3.2. Additional Dietary Interventions

Other dietary modifications that affect genome stability in mice have been identified, though the evidence remains limited. Both short-term fasting and fasting-mimicking diets reduce caloric intake and are suggested to trigger protective mechanisms similar to those found in DR. Both diet types have been associated with a range of beneficial health outcomes, including enhanced stem cell regeneration and reduced inflammation across various organisms [77,78,79,80]. Likewise, alternate-day fasting reduced spindle structure abnormalities and chromosome segregation errors while restoring antioxidant defense capacity and eliminating excess reactive oxygen species (ROSs) [81]. Furthermore, fasting for a single day has been observed to improve survival rates, accompanied by increased expression of various DNA repair genes and a reduction in γH2AX foci in small intestinal epithelial stem cells, following a dose of the DNA-damaging chemotherapeutic etoposide that is lethal to fed littermates [82,83]. Together, these findings suggest that fasting, like CR/DR, may have genome-protective effects, consistent with previous reports regarding improved health in mice and other organisms [84].

Similarly, low glycemic index (GI) diets, which prioritizes the consumption of foods that induce a slower and more gradual release of glucose into the bloodstream, have been associated with improved health, likely by improving lipid metabolism and lowering postprandial hyperglycemia-associated ROS [85,86,87]. For instance, a study with wild-type Balb/c mice demonstrated that implementing a low GI diet late in life (20 months of age) still extended median lifespan by 12% while reducing plasma glucose levels, abasic site frequency, and levels of the DD proxy 8-OHdG [88]. Importantly, the average food intake remained constant while digestible energy was only 3.5% lower in the GI diets, thus minimizing confounding effects due to CR/DR-like effects.

Macronutrients (carbohydrates, proteins, and fats) play an important role in shaping the dietary impact on health and disease. While high-fat diets (without ketone induction) are often utilized to investigate the harmful effects of obesity, modifications in dietary protein, such as the restriction of specific amino acids, enhanced both health and lifespan in wild-type mice [89,90,91]. For instance, methionine restriction in progeroid Hutchinson–Gilford progeria syndrome (HGPS) mutant mice extended median and maximal lifespan, and reversed transcriptomic alterations linked to inflammation and DNA repair pathways [92]. These beneficial effects are likely mediated by metabolic changes, as several associated pathways, including fatty acid metabolism, were also normalized [92]. This is further supported by studies in *Ercc1*^Δ/−^ mutant and wild-type mice indicating that adjusting macronutrient ratios, particularly the balance between carbohydrates and proteins, can enhance metabolic health and aging outcomes [30,93,94].

Curiously, despite the beneficial effects associated with protein restriction, an increase in protein intake through twice-daily supplementation of whey peptides (200–400 mg/kg) in wild-type mice also yielded positive outcomes. This supplementation protected against UVB irradiation-induced photoaging, as indicated by reduced cutaneous thickening, wrinkle formation, and melanin granules, while also preserving cutaneous elasticity, likely by reducing levels of collagen-degrading metalloproteinase-2 [95]. Furthermore, whey peptide supplementation significantly decreased the presence of Ki-67 and 8-OHdG-positive cells in the stratum basale of these chronically UVB-irradiated mice, indicating a potential role in mitigating cellular proliferation and oxidative DD, potentially mediated by VEGF [95]. While protein intake is generally associated with reduced food intake [96], the study above did not report on food intake or whey peptide-induced skin changes without chronic UVB irradiation, warranting caution and further investigation into the effects of whey peptides on genomic stability.

Integrating findings from the broader literature, we propose a (simplified) scenario for the effects of various forms of dietary interventions, particularly CR/DR, on lifespan and health, at least in part by influencing DD and DNA repair mechanisms, as presented in Figure 2, while recognizing interactive mechanisms, such as hormesis-induced resilience, likely modulate these processes both directly and indirectly. Several of the above-indicated diets overall reduce the proliferation rate of healthy body cells, which distinctively affects replication-dependent DNA repair processes like HR and XLR, as well as replication-independent DNA repair processes such as TCR and NHEJ. Combined with the suppression of metabolic activity and ROS levels, this results in a global reduction in genomic instability, lowering the incidence of cells undergoing apoptosis or entering a state of cellular senescence, preventing systemic inflammaging and functional decline in tissues [25,26,27,35,41,45,46,49,51,52,56,61,64,69,71,72,73,74,75,76] (Figure 2). Furthermore, factors like sex, genotype, and the specific type of dietary intervention likely influence efficacy as well, with variations in duration, meal timing, and intensity further modulating these outcomes [44,48,97,98]. Future research should examine the influence of these factors and move beyond mere correlation by directly assessing DD markers under diverse conditions to determine the causal role of these interventions on genomic stability and longevity.

### 3.3. Prebiotics and Probiotics

The impact of diet on the microbiota is well-documented, with numerous studies highlighting how dietary choices shape microbial communities [99,100,101]. Notably, aging-associated physiological changes (e.g., immune system decline and intestinal permeability) can shift the microbiota towards a less diverse and more pro-inflammatory composition, which has been associated with both inflammaging and frailty, further accelerating this shift [102,103]. Furthermore, this shift causally contributes to increased levels of γH2AX in the liver and heart through inhibition of DD repair and decreased antioxidant capacity [104,105], suggesting that reversal of microbiota composition through probiotic interventions might improve genomic stability.

Recent work supports this notion, where supplementation with the prebiotic neoagarotetraose in aged mice extended lifespan, reduced neuronal DD, and shifted the gut microbiota toward a more youthful, health-associated composition, resulting in increased short-chain fatty acid production and reduced neuroinflammation [106]. Similarly, specific probiotic strains such as Bifidobacterium bifidum BGN4 and Bifidobacterium longum BORI were found to reduce the number of γH2AX positive neurons in the hippocampus of aged wild-type mice [107] ‡. In another study, supplementation with Lactobacillus casei strain Shirota (LcS) ameliorated sarcopenia in SAMP8 mutant mice, a senescence-accelerated model with increased DD, by reconfiguring the microbiome and upregulating the production of short-chain fatty acids, resulting in decreased expression of apoptotic, p53 signaling, NHEJ, and inflammatory pathways in muscle tissue [108].

Additionally, early-life colonization of wild-type mice with *Lactobacillus rhamnosus* GG was associated with activation of the protective *Sirt1*/*Ampk*/*Ppargc1a* pathway, which coincided with enhanced antioxidant defenses (*Sod1* and *Sod2* upregulation) and reduced markers of DD (γH2AX) and inflammation (*Il1a*, *Il6*, *Tnf*) in the colon [109]. Furthermore, in DNA repair-deficient *Ercc1*^Δ/−^ mutant mice, supplementation with *Akkermansia muciniphila* mitigated age-related thinning of the colonic mucus layer and decreased the expression of pro-inflammatory genes in the colon [110]. While these findings suggest a potential mechanistic link, the precise causal contributions of microbiota-derived factors, such as short-chain fatty acids, remain to be fully elucidated [109].

Collectively, these findings suggest several potential mechanisms by which the microbiota may influence host genomic stability. Beyond the observed modulation of inflammatory responses and enhancement of antioxidant defenses, other mechanisms may include the microbiota’s influence on host metabolism and nutrient absorption, affecting the availability of essential cofactors for DNA repair enzymes [111,112]. While further research is needed to fully elucidate these mechanisms and exclude artificial explanations, such as multiple-testing artifacts, the emerging evidence suggests that targeted modulation of the gut microbiota through probiotic interventions or dietary changes could be a promising strategy to promote genomic stability.

### 3.4. Micronutrients

Maintaining essential micronutrients within specific physiological ranges is crucial for optimal cellular function and the prevention of adverse health outcomes, while deviations from these ranges can lead be permanent detrimental outcomes [113,114,115]. Despite extensive research documenting the impact of micronutrient deficiencies on health [116,117,118], the precise extent of these optimal dose ranges and their impact on genomic stability in mice remain poorly characterized.

Most research investigating the role of micronutrients in genomic stability in mice primarily focuses on their antioxidant potential. For instance, vitamin C supplementation has been shown to exert varying effects on DNA integrity depending on environmental and genetic context, with benefits observed in Werner syndrome progeroid mutant mice (*Wrn*^Δhel/Δhel^) and wild-type C57BL/6J mice, while vitamin C supplementation had no effect on wild-type mice under cold stress conditions [119,120,121]. Similarly, studies on vitamin E supplementation have yielded mixed results. In DNA repair-deficient progeroid *Xpg*^−/−^ mutant mice, vitamin E has been associated with a reduction in the number of TRP53-positive cells and an extension of neurological healthspan [122] ‡. However, other studies have reported no significant effect of vitamin E on oxidative DD in wild-type mice [123,124], although vitamin E was observed to increase both median and maximum lifespan, likely through enhanced resilience mechanisms [124]. Adding to the growing evidence that micronutrients may affect the genome, recent data show that folic acid, a vitamin B9 precursor, can mitigate radiation-induced lung injury by suppressing senescence-associated signaling [125].

Multi-micronutrient supplementation has shown promise in enhancing genomic stability and mitigating DD. Studies have reported increased long-term survival following radiation or genotoxic exposure [126,127], reduced apoptosis in *Apoe*^−/−^ mutant mice on a high-fat diet [128], and lower levels of malondialdehyde and 8-OHdG oxidative stress markers in prematurely aging mutant mice [129].

Based on the studies reviewed, the potential benefits of optimizing micronutrient dosages beyond adequate levels remain unclear. When present at adequate levels, micronutrients appear to delay age-associated metabolic changes and reduce genomic instability. However, to what extent are these effects attributable to antioxidant activity or metabolic regulation, rather than alternative mechanisms, is currently largely unknown [130]. Beyond direct redox actions, micronutrients may indirectly influence genome integrity by modulating cellular stress responses, metabolic homeostasis, or damage tolerance pathways in a compound-specific manner. Such effects could alter either the burden of DNA damage or its downstream consequences, highlighting the need for further research in this area.

### 3.5. Fatty Acids

Recent work highlights the role of fatty acids, specifically omega-3 polyunsaturated fatty acids (*n*-3 PUFAs), in the context of DD and repair. Docosahexaenoic acid (DHA) is a major *n*-3 PUFA found in fish oil, which plays a pivotal role in reducing oxidative stress and enhancing genomic stability. This effect is mediated through the upregulation of antioxidant enzymes SOD1, SOD2, and catalase in the liver and heart, thereby reducing oxidative stress markers and, e.g., protecting against telomere shortening [131]. DHA also alleviates cellular senescence and ameliorates inflammation and γH2AX accumulation through improved mitochondrial homeostasis and recruitment of PARP1, a key component in the DD response, in telomerase-deficient mutant mice [132].

Additionally, maternal diets rich in *n*-3 PUFAs have been proposed to enhance homeostatic control, leading to the suppression of aberrant cell proliferation in the F1 offspring of breast cancer-prone mutant mice. This improved homeostatic regulation is associated with upregulation of pathways related to p53 signaling, DNA repair, and inflammatory responses [133]. Notably, while *n*-3 PUFAs appear beneficial in wild-type mice, a study with *Ercc1*^−/−^ mutant mice demonstrated that a diet high in safflower oil, consisting of ~90% *n*-6 and *n*-9 PUFAs [134], actually shortened lifespan and increased γH2AX in vitro [135], suggesting a potential hormetic or PUFA-specific effect on DD.

Extra virgin olive oil (EVOO), a rich source of monounsaturated fatty acids, has been associated with enhanced genome stability by counteracting hypomethylation induced by environmental carcinogens such as dimethylbenz[a]anthracene and trifluoroacetic acid. This protection may help suppress hypomethylation-driven LINE-1 retrotransposon activity, a class of mutagenic non-coding DNA [136,137]. The current number of mechanistic studies is, however, limited, and further research is needed to characterize the involved molecular compounds and validate these effects more broadly.

In conclusion, fatty acids, particularly *n*-3 PUFAs and monounsaturated fatty acids, exhibit potential in promoting genomic stability and reducing DD, in contrast to *n*-6 and *n*-9 PUFAs, which might be able to increase DD. These effects are likely achieved through oxidative stress reduction, telomere preservation, and cellular senescence alleviation. However, the varied impact of different PUFAs on DD pathways highlights the complexity and potential specificity of these interactions, necessitating further research to fully understand these mechanisms in relation to genome stability.

### 3.6. Hormones

Diet influences many somatic processes, including hormone levels. The importance of hormones in somatic functions has long been recognized, and there is growing evidence that hormone regulation through supplementation can play a role in maintaining genomic stability and mitigating age-related decline. For example, supplementation with dehydroepiandrosterone (DHEA), a steroid hormone precursor and ligand of PPARα, PXR, and CAR [138], delays the age-associated decline in oocyte cohesin levels and reduces the level of γH2AX foci in aged wild-type C57BL/6J mice [139]. This improved chromosomal stability correlates with decreased oocyte apoptosis and amelioration of follicle count, ovarian aging, and fertility decline [139].

Melatonin, a sleep–wake cycle synchronizing hormone and potent antioxidant whose production diminishes with aging, shows promising effects on DNA repair mechanisms in a supplementation time-dependent manner. In aged wild-type Swiss albino mice, eighteen months of melatonin supplementation starting at three months of age increased lifespan, reduced DD markers, and increased the levels of APE1 and OGG1 repair enzymes, indicating an enhanced DNA repair capacity [140]. This effect may be attributed to melatonin’s ability to modulate circadian rhythms and sleep patterns, which also influence cellular repair processes [141,142]. However, it is unknown how well this treatment would translate to other mouse models, as Swiss mice exhibit a 94% reduction in pineal gland melatonin levels, but only a 25% reduction in plasma when compared to wild-type C3H/HENHSD mice [143], complicating the interpretation of these findings.

### 3.7. Nicotinamide Adenine Dinucleotide Precursors

One process closely regulated by the circadian rhythm is the synthesis of nicotinamide adenine dinucleotide (NAD+), a vital coenzyme that plays a critical role in numerous cellular processes, including energy metabolism, DNA repair, and epigenetic regulation. However, with aging, NAD+ levels decline due to several factors, including senescence-associated CD38 activation, increased consumption by poly(ADP-ribose) polymerases (PARPs) in response to DD, and declining biosynthesis efficiency [144,145,146]. To counteract this age-related decline, supplementation with precursors such as nicotinamide (NA), nicotinamide mononucleotide (NMN), and nicotinamide riboside (NR) has been proposed. These precursors boost NAD+ availability and could thus enhance cellular repair and overall cellular health. Importantly, reported health benefits of NAD+ precursors can reflect both NAD+-dependent DD signaling and/or repair and indirect effects via improved mitochondrial function, redox homeostasis, and inflammatory signaling that may reduce the upstream burden of DD.

One of the key consumers of NAD+ is sirtuins, a class of NAD+-dependent deacetylases important for various processes, including metabolism, inflammation, and apoptosis [147,148,149,150]. Supplementation with NAD+ precursors have, likely due to its central role across various cellular pathways, shown promise in various disease models. In a mouse model of alcohol-associated liver disease, NA lowered the expression of DD marker genes (e.g., *Rec8* and *E2f1*) while upregulating *Sirt1* [151]. Likewise, in a D-galactose-induced aging model, NMN improved locomotor activity and spatial memory, attenuated oxidative stress, neuroinflammation, and apoptosis, and preserved intestinal barrier integrity. Notably, these benefits were abolished by pharmacological SIRT1 inhibition, underscoring the importance of SIRT1 [152]. In Alzheimer’s disease models, NR supplementation has been reported to effectively decrease microglia and astrocyte activation and reduce neuroinflammation, senescence markers, and apoptosis in hippocampal neurons, while also promoting mitophagy, synaptic function, and cognition [153,154,155]. Complementing these findings, NR modulates brain region-specific redox and metabolic networks, enhancing mitochondrial pathways in the cerebral cortex and neurotransmitter regulation in the hippocampus [156]. In the cardiovascular system of *Snhg12*^−/−^ mutant mice, a model of atherosclerotic lesion formation characterized by DD and senescence, NR significantly abrogated γH2AX foci formation and related senescence markers, an effect that may reflect reduced damage accumulation and/or altered damage signaling [157]. Some of these beneficial effects extend to DNA-repair-deficient models of accelerated aging like *Csb*^−/−^ and *Ercc1*^∆/−^ mutant mice, where NR supplementation sustains SIRT1 activity, maintains mitochondrial function, and partially ameliorates their progeroid phenotypes [158,159], despite their inherently lowered DNA repair capacity, suggesting that these health benefits likely arise from mechanisms distinct from direct enhancement of repair.

NR has also demonstrated potential in improving genomic stability in *Tert*^−/−^ mutant models by preserving telomere length and attenuating 53BP1-associated DD signaling. This leads to enhanced mitochondrial function and reduced systemic inflammation, ultimately mitigating hematopoietic myeloid skewing [160] ‡. Another NAD+ supplement, NMN, has also shown beneficial effects in *Tert*^−/−^ mice, as it reduced liver fibrosis, an effect that appears to be partially dependent on SIRT1 activation [161] ‡. Additionally, NMN can rejuvenate mitochondrial function in aged oocytes of healthy wild-type mice, resulting in improved redox homeostasis, reduced endogenous γH2AX foci, and normalized spindle and chromosome structure [162,163].

These studies, along with others that supplement NAD+ precursors through injection [164,165,166,167,168,169], collectively highlight the broad-ranging benefits of NAD+ precursors in counteracting age-related cellular decline. While direct stimulation of NAD^+^-dependent DNA repair pathways may contribute, reductions in DD markers are likely mediated indirectly through improved mitochondrial function and the consequent reduction in endogenous damage burden. Distinguishing direct repair enhancement from secondary protective effects in vivo remains an important objective for future research. Overall, by supporting metabolic health and enhancing cellular resilience against DD, NAD+ precursors show promise as therapeutic agents for promoting longevity and mitigating the effects of aging and diseases associated with genomic instability.

### 3.8. Plant Derivatives

A substantial number of plant-derived compounds have been reported to enhance genomic stability. However, many of these compounds have not been comprehensively studied in relation to DD in mice. Nonetheless, a large proportion is reported to exhibit common biological effects like enhanced mitochondrial function, reduced inflammation, or the induction of cellular stress responses, thereby promoting genomic stability, immune function, and fertility [170,171,172,173,174,175,176,177,178,179,180,181,182,183,184,185,186,187,188,189] ‡.

Additionally, some plant derivatives have demonstrated protective effects against exogenous sources of DD. For example, theaflavins [190], carotenoids [191], and several plant extracts and compound derivates [192,193,194,195,196,197,198] ‡ have been shown to possess radioprotective properties, mitigating DD and related effects induced by UVB and gamma radiation. Furthermore, theaflavins [199] and Shoutai pills [200] have also been reported to confer protection against DD induced by chemotherapy, as evidenced by reduced γH2AX levels in wild-type mice. Some plant-derived substances have also shown efficacy in mitigating genomic insults induced by injury [201,202], exposure to genotoxic chemicals [203,204], or iron-induced oxidative stress [205]. Other compounds have exhibited anticarcinogenic properties in cancer-prone contexts [206,207] ‡ or in carcinogen-related causes of cancer [208,209], highlighting the potential of plant derivatives not only to prevent DD but also to mitigate its long-term consequences across various contexts of genomic stress.

While many studies are fragmentary and the efficacy and precise mechanisms of many of these plant-derived substances in DD and repair are incompletely understood, the consistency in their downstream effects, e.g., enhanced cellular resilience and improved DNA repair, lends credibility to their potential therapeutic value. Further investigation into these shared mechanisms could reveal broader applications for DNA repair and cellular health, potentially leading to the development of novel therapeutic strategies based on these natural compounds.

### 3.9. Synthetic Drugs

Natural compounds have frequently inspired the development of synthetic drugs, which, in some cases, also exhibit potential in enhancing genomic stability and modulating the effects of natural aging. These synthetic compounds exert their influence through mechanisms similar to their natural counterparts, including the regulation of energy metabolism, resilience, and inflammation.

For instance, the oral administration of the antioxidant butylated hydroxyanisole, as well as the antioxidant inducer oltipraz, have been demonstrated to reduce endogenous single-strand DD in the liver during aging of wild-type Swiss-Webster mice, likely through the preservation of glutathione levels [210]. Similarly, metformin, a widely used antidiabetic drug, has been found to decrease ROS production, upregulate antioxidant enzymes, and inhibit γH2AX and cellular senescence in various disease models [211,212,213,214].

Beyond general antioxidant and metabolic effects, several synthetic compounds have demonstrated specific interactions with distinct cellular processes that contribute to genomic stability and the modulation of aging [215,216,217,218] ‡. For instance, the small-molecule DDO1002, an NRF2–KEAP1 PPI inhibitor, lowered intracellular ROS and reduced the DD marker γH2AX, delayed cellular senescence, and improved regenerative capacity in wild-type C57BL/6J aged or irradiated hematopoietic stem cells [218]. Another example is aspirin and its nitric oxide-donating derivative, which mitigate microsatellite instability in a Lynch syndrome mouse model deficient in mismatch repair (MMR), leading to increased lifespan through the modulation of stress-related signaling pathways [219]. Low-dose aspirin likewise showed a tendency toward extended lifespan in DNA repair-deficient *Xpg*^−/−^ mutant mice (*p* = 0.056) [159]. Exogenous protection has also been reported. The fatty acid oxidation inhibitor meldonium reduces mitochondrial DD and improves cognitive function in aged wild-type mice subjected to LPS-induced inflammation [220]. The senolytic compound ABT-263, a Bcl-2/Bcl-xL inhibitor, selectively eliminated doxorubicin-induced senescent retinal pigment epithelial cells and alleviated retinal degeneration in wild-type mice, while reducing CDKN1A (p21) and CDKN2A (p16) protein levels, suggesting mitigation of DD-associated senescence [221].

In mutant models with deficient DNA repair accelerated aging, synthetic compounds have also demonstrated significant benefits. In *Ercc1*^∆/−^ mice, treatment with the HSP90 inhibitor and senolytic agent 17-DMAG reduced *Cdkn2a* expression and cellular senescence [222] ‡. Additionally, the compound UCM-13207 has been found to extend lifespan and improve healthspan in HGPS models by reducing nuclear levels of progerin, thereby decreasing senescence and the γH2AX DD markers associated with it [223]. Interestingly, since progerin levels also gradually increase during natural aging, it may be worthwhile to validate this treatment in wild-type models.

While these findings are promising, most of these substances have primarily been studied in specific contexts characterized by heightened DD or impaired repair capacity. Further research is needed to fully elucidate the potential of these compounds in mitigating genome instability-driven aging across broader contexts and conditions.

### 3.10. Miscellaneous Compounds and Interventions

A variety of substances, not classified under the earlier categories used in this study, along with combinations of previously discussed interventions [224,225], have shown potential in enhancing genomic stability. Many of these compounds, like hydrolyzed chicken extract ‡ and royal jelly, modulate ROS homeostasis similarly to other treatments previously examined [226,227]. This modulation can result in a decrease in DD markers, like γH2AX and 8-OHdG, following genomic insults [227,228,229,230], while some of these interventions also prolong the lifespan of wild-type and *Atm*-deficient mutant mice [226,231,232]. However, the relationship between these substances and genome stability in mice has often been explored in only a single study, limiting the generalizability of these findings beyond their specific experimental conditions. For example, spermidine has recently been recognized as a pro-longevity drug that protects wild-type C57BL/6J mice against the genotoxic and mutagenic compound 4-nitroquinoline 1-oxide, resulting in a slowdown of oral carcinoma development and a reduction in DD, as measured by γH2AX and comet assay tail moment [233].

Another compound, taurine, a semi-essential amino sulfonic acid, has recently been identified to influence aging. Circulating taurine levels decline with age in mice, monkeys, and humans, and supplementation was shown to reduce DD, cellular senescence, and mitochondrial dysfunction while extending lifespan in various organisms, including wild-type mice [234].

Another relatively well-documented substance is pyrroloquinoline quinone (PQQ), a vitamin-like biofactor that has shown to exert genome-protective effects in various situations of increased genomic instability [235]. *Bmi*^−/−^ mutant mice exhibit increased oxidative damage, reduced cell proliferation, increased cell senescence, and compromised collagen synthesis due to enhanced matrix metalloproteinase activity. PQQ supplementation alleviated these pathological changes and reduced oxidative damage and DD, as indicated by decreased γH2AX markers [236,237]. In osteoporosis-prone orchiectomy mice, PQQ lowered the DD-related markers γH2AX, TRP53, CHK2, and NFκB p65 [238]. Similarly, in natural aging-related osteoporosis in wild-type mice, PQQ supplementation reduced oxidative stress, osteocyte senescence, and DD (8-OHdG immunostaining) while preventing bone loss. This involved stabilization of MCM3 and activation of the Keap1-Nrf2 axis, which strengthened antioxidant defenses and reduced DD in osteoblasts [239]. In a model of osteoarthritis induced by anterior cruciate ligament transection, PQQ treatment reduced DD, inflammation, and senescence in articular cartilage compared to sham-operated controls [240], suggesting a potential role in genomic stability and longevity.

In conclusion, various compounds, such as royal jelly and PQQ, show promise in enhancing genomic stability and reducing DD markers. While these interventions have demonstrated benefits under specific experimental conditions, the limited number of studies in relation to natural aging calls for further research to validate their broader applicability. Expanding research in this area could pave the way for novel therapeutic strategies aimed at preserving genome integrity and promoting healthy aging.

## 4. Discussion

**Figure 3:**
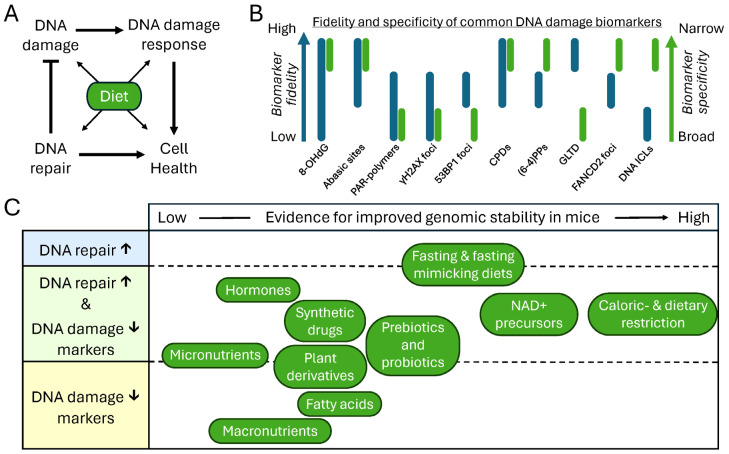
Schematic representation of consumable interventions and their impact on DNA damage and repair. (**A**) Simplified schematic depicting the relationships between DNA damage burden, DNA repair, DNA damage response signaling, and cellular health, which all could be affected by dietary interventions. Note that by altering metabolism, diet could influence DNA damage bidirectionally. (**B**) Relative technique-dependent detection fidelity (blue) and biomarker specificity (green) of commonly used DNA damage markers. Bar ranges reflect variability among measurement techniques and biomarker specificity (also see details on biomarker specificity and accuracy/reliability of the detection method in Table 1). (**C**) Interventions are categorized into three groups: those that increase DNA repair (**top**), those that reduce DNA damage (**bottom**), and those that have dual effects (**middle**). The horizontal positioning of interventions reflects the strength of evidence supporting their effects, with those on the right, such as CR/DR, being more strongly supported by evidence than those on the left, which in contrast mostly consisted of interventions based on a few studies, sometimes using only a single marker.

Aging is affected by various factors. One of these is dietary consumption, which influences homeostasis by modulating energy balance, metabolic fitness, and overall health. Alterations to these processes, e.g., by excessive food intake [26], are closely linked to aging and age-related diseases, with DD being a significant mediator, as shown by DNA repair-deficient syndromes and progeroid mouse models. In this review, we highlighted and assessed the evidence of known nutritional interventions that improve genomic stability and healthy aging in mice. Many interventions discussed here, particularly CR/DR and their mimetics, act on more than one mechanistic layer (e.g., different DNA repair processes, metabolism, and/or antioxidant systems; see also Figure 2), complicating causal interpretation when relying solely on DD markers or lifespan readouts. Moreover, while some dietary interventions have been studied for decades, others are supported by only a limited number of small studies, and relatively few directly assess DD or DNA repair. In Figure 3, we outline the relationship between DNA damage, response signaling, and repair, and their effects on resilience; summarize the relative fidelity and specificity of common biomarkers; and provide a qualitative contextual overview of the discussed literature. Interventions were positioned based on the amount and consistency of available data, and on how the reported outcomes relate to genomic stability (DD markers and/or repair readouts). As such, Figure 3 should be viewed as a guide to where evidence is stronger and where important gaps remain.

Limitations of this study include the lack of a formal quality assessment, as usually indicated in systematic reviews. Instead, our semi-systematic search resulted in a broad overview of the literature, for which we acknowledge a potential bias due to high heterogeneity between studies (i.e., due to varying mouse strains, tissues, intervention lengths, and markers) and/or a small number of studies per compound, making reproducibility unclear. Such compounds/interventions with limited studies are therefore also more depicted on the left side of Figure 3. Concomitantly, tissue-specific effects, strain variability, or sex differences are not discussed or only marginally indicated, due to the limited number of studies identified for several dietary interventions, particularly plant derivatives and synthetic drugs, which often assessed only a single or less specific biomarker, limiting generalizability. However, we do refer to such reviews with a focus on the most robust health- and lifespan-extending intervention: calorie restriction [34,44,48]. Moreover, this review was intended to reveal interventions that could enhance genomic stability and to serve as guide for future longevity research. As such, we revealed many connections of nutrition-based interventions affecting genome integrity for further exploration.

The restriction of diet (CR/DR), which is known to robustly delay aging in a wide range of organisms separated by large evolutionary distances, from mice to non-human primates, indicating that its underlying molecular mechanism is strongly conserved, is emerging as a genuine, potent modulator of DD, repair, and response processes in various organisms [14,45,51,241]. Besides extending lifespan and improving genomic stability through the above-indicated mechanisms, CR (mimetics) influence multiple age-related nodes, including epigenetic integrity, autophagy, and activation of cellular stress responses [242,243,244,245]. Thus, genome maintenance appears to be part of a coordinated resilience program aiming to promote cellular and organismal survival at the expense of growth.

Beyond model systems, CR and related strategies such as intermittent fasting are being explored in oncology and neurodegeneration, with early trials indicating feasibility and acceptable safety. However, efficacy in humans remains inconclusive, potentially reflecting adherence challenges, mismatches with standard-of-care regimens, or biological differences [245,246,247,248,249]. Species-specific physiological features, such as higher mass-specific metabolic rates, shorter lifespans, and accelerated molecular turnover in mice, are likely to shape both damage accumulation and responsiveness to intervention. These differences suggest that dietary interventions that robustly extend lifespan or reduce DD in mice may require careful adjustment in timing, duration, or intensity to achieve benefits in humans.

Interestingly, many of the interventions discussed here act on a subset of mechanisms employed by CR/DR, hence operating as partial mimetics without necessitating reduced food intake [250]. NAD^+^ precursors such as NMN and NR, for example, activate sirtuins and improve mitochondrial function, recapitulating some CR/DR-associated benefits; however, current evidence suggests that their effects on genomic stability are largely indirect, arising from improved metabolic homeostasis, reduced inflammation, and enhanced cellular stress resilience rather than from direct stimulation of DNA repair [251,252]. Another emerging class of metabolic modulators includes GLP-1R and GIPR agonists, such as semaglutide and tirzepatide, which reduce appetite, lower body weight, and counteract metabolic diseases like diabetes. While their primary mechanisms involve metabolic regulation, their potential effects on genomic stability and aging remain underexplored. Given their strong influence on energy balance and cellular stress pathways, future studies should assess whether these compounds also contribute to DD mitigation or repair processes, potentially offering novel avenues for aging interventions.

However, while some interventions demonstrate promising results, their effects are often less pronounced or process-specific. To remedy this, future research should focus on exploring combinations of dissimilar interventions to identify optimal strategies for mitigating DD and promoting longevity [253,254]. Another aspect that should be considered is that basal genotoxic exposure, energy expenditure, and cell division rates vary between tissues, leading to non-uniform aging that might influence the organ-specific efficacy of these interventions.

Currently, much of the research in this field is rather fragmented or inconsistent, with highly variable techniques like the comet assay and frequent reliance on RNA expression data without confirming protein levels or functional impact on DNA repair. Establishing rigorous markers for assessing DD and genome instability, such as γH2AX foci, 53BP1, or RAD51 foci, and novel nanopore-based structural analysis for DNA damage [255], along with standardized protocols, would greatly enhance study reproducibility and comparability. Without such consistency, drawing reliable conclusions about the effectiveness of nutritional interventions remains challenging.

Beyond methodological inconsistencies, another challenge lies in testing these findings in a real-world setting. Laboratory experiments often focus on endogenous or supraphysiological levels of exogenous DD, while real-world conditions present additional challenges from environmental stressors. Future studies should incorporate realistic environmental insults to genome stability to better assess the efficacy of interventions. Moreover, the issue of survival bias in publishing needs consideration, as positive results are much more likely to be published, skewing the perceived efficacy of interventions. Potential translation to humans is additionally complicated because some interventions are mentally exhaustive (CR/DR), expensive (synthetic diet to limit specific amino acids), or prone to interfere with social aspects of life (fasting). In contrast, other interventions like NAD+ precursors or oral medication are quite feasible and are being actively trialed in humans [256,257,258].

One key area of debate in this context is the role of oxidative stress and antioxidants in genomic stability. While many studies focus on the antioxidative properties of a substance, evidence that ROS at physiological levels causes genomic damage [259,260,261], or that antioxidants are beneficial protectors of the genome [262,263,264], is heavily debated. Moreover, studies in humans have often yielded negative results, with various meta-analyses even suggesting a likely increase in disease incidence [265,266]. While the exact reasons for this are unknown, dosage and context might be an important factor, highlighting the limited ability of in vitro antioxidant assays to predict in vivo efficacy [267]. For example, while physiological levels of vitamin C behave largely as an antioxidant in vivo, pharmacological dosing can shift toward prooxidant activity via Fenton chemistry. Moreover, although the increase in oxidative DD and engagement of DNA repair pathways can be beneficial in some situations (e.g., cancer treatment), it does show the importance of situational awareness and balanced application of these and similar types of compounds [268,269]. Additionally, the genome-protective effects of many so-called “antioxidative” compounds extend beyond simple ROS scavenging, as they often also modulate stress resilience pathways and energy metabolism to improve cellular function, longevity, and genomic maintenance [270,271,272].

Alternatively, compounds that boost endogenous antioxidant systems (e.g., as associated with fasting and DR) might result in a stronger and more adjusted response than the artificial administration of exogenous antioxidants. The optimal timing and duration of interventions targeting ROS and genomic stability also warrant consideration. While some approaches may require lifelong implementation (e.g., ROS scavengers), there is a growing interest in compounds that potentiate long-term effects to improve efficacy and reduce costs. Elucidating these aspects will therefore be crucial for developing targeted therapeutic applications.

## 5. Conclusions

In conclusion, the modulation of genomic instability by consumable interventions holds considerable promise, but future research should broaden their focus beyond antioxidant mechanisms and extend the scope of investigation to include the effects of these interventions on the natural aging process. Moreover, combinatory approaches may yield synergistic benefits, leading to a deeper understanding of the underlying mechanisms and the development of more comprehensive strategies for promoting genomic stability and healthy aging.

## Figures and Tables

**Table 1:** Major types of DNA damage and their predominant characteristics [12,13,14,15,16,17,18,19,20,21,22,23,24,25].

	Base Modifications	SSBs	DSBs	Helix-Distorting Lesions	Interstrand Crosslinks
**Causes**	Deamination, ROS, alkylation, and UV radiation	ROS, ionizing radiation, and topoisomerase I inhibitors	Ionizing radiation, replication fork collapse, and topoisomerase II inhibitors	UV radiation, polycyclic aromatic hydrocarbons, ROS, and chemotherapeutics	Endogenous aldehydes and chemotherapy
**Consequences**	Mutations and altered epigenetic regulation	Replication-induced DSBs	Chromosomal aberrations, cell cycle arrest, apoptosis, and senescence	Transcription and replication stalling, mutagenesis, and cell death	Transcription and replication stalling, cell cycle arrest, cell death, and senescence
**Repair** **Mechanisms**	BER and direct reversal	SSBR (specialized BER)	NHEJ, HR, alt-EJ, and single-strand annealing	NER and TCR	FA pathway, NER, and HR
**Biomarker** **Examples ^$^**	8-OHdG, N7-methylguanine (alkylation), and abasic (AP) sites	XRCC1 foci and PAR-polymers ^$^	γH2AX foci ^$^, 53BP1 foci ^$^, RAD51 foci ^$^, and ATM/ATR activation ^$^	CPDs, (6-4) photoproducts, R-loops ^$^, and GLTD ^$^	FANCD2 foci and DNA interstrand crosslinks
**Detection** **Methods ^1–3^**	ELISA ^2^, mass spectrometry ^1^, IF ^2^, IHC ^2^, and comet assay (FpG, Endo III) ^3^	IF ^2^, alkaline comet assay ^3^, and FADU assay ^3^	IF ^2^, IHC ^2^, Western blot ^2/3^, neutral comet assay ^3^, pulsed-field gel electrophoresis ^2^, and FADU assay ^3^	IF ^2^, IHC ^2^, sequencing ^1^, UDS assay ^1^, Recovery RNA/DNA synthesis ^1^, and GLTD ^1/2^	IF ^2^ and crosslinking comet assay ^3^
Effect on Aging *	+	++	+++	+++	++

SSBs = single-strand breaks, DSBs = double-strand breaks, ROS = reactive oxygen species, 8-OHdG = 8-hydroxyguanosine, ELISA = enzyme-linked immunosorbent assay, FpG = formamidopyrimidine DNA glycosylase, IHC = immunohistochemistry, IF = immunofluorescence, FADU = fluorometric analysis of DNA unwinding, Endo = endonuclease, BER = base excision repair, SSBR = SSB repair (specialized BER), NHEJ = non-homologous end joining, HR = homologous recombination, alt-EJ = alternative end joining, NER = nucleotide excision repair, TCR = transcription-coupled repair, FA = Fanconi anemia, CPDs = cyclobutane pyrimidine dimers, GLTD = gene length-dependent transcriptional decline, UDS = unscheduled DNA synthesis. ^$^ DNA damage biomarkers that have broad specificity or can be secondary to other lesions. ^1–3^ degree of accuracy/reliability of the detection method (1 high; 2 intermediate or depending on antibody specificity; 3 lower/variable). * pluses indicate an estimated relative contribution of each lesion type to aging (weak (+), moderate (++), and strong (+++) effects), inferred from available experimental and clinical evidence.

## Data Availability

The original contributions presented in this study are included in the article/Supplementary Material. Further inquiries can be directed to the corresponding author.

## Supplementary Materials

The following supporting information can be downloaded at: https://www.mdpi.com/article/10.3390/nu18020246/s1, File S1: Keywords and search query; File S2: PubMed references search 1 (till March 2023); File S3: PubMed references search 2 (2023–2025).

## Abbreviations

The following abbreviations are used in this manuscript:

DD	DNA damage
BER	Base excision repair
NER	Nucleotide excision repair
TCR	Transcription-coupled repair
HR	Homologous recombination
NHEJ	Non-homologous end joining
DR	Dietary restriction
CR	Caloric restriction
IGF1	Insulin-like growth factor
ICL	Interstrand crosslink
DSB	Double-strand break
8-OHdG	8-Hydroxyguanosine
UDS	Unscheduled DNA synthesis
GG-NER	Global genome nucleotide excision repair
XLR	Crosslink repair
GLTD	Gene length-dependent transcriptional decline
HSCs	Hematopoietic stem cells
ROS	Reactive oxygen species
GI	Glycemic index
HGPS	Hutchinson–Gilford progeria syndrome
PUFAs	Polyunsaturated fatty acids
DHA	Docosahexaenoic acid
EVOO	Extra virgin olive oil
DHEA	Dehydroepiandrosterone
NAD	Nicotinamide adenine dinucleotide
PARP	Poly ADP-ribose polymerase
NA	Nicotinamide
NMN	Nicotinamide mononucleotide
NR	Nicotinamide riboside
MMR	Mismatch repair
PQQ	Pyrroloquinoline quinone
